# Standards of lithium monitoring in mental health trusts in the UK

**DOI:** 10.1186/1471-244X-10-80

**Published:** 2010-10-12

**Authors:** Noel Collins, Thomas RE Barnes, Amber Shingleton-Smith, David Gerrett, Carol Paton

**Affiliations:** 1 Central and North West London Foundation Trust, Greater London House, Hampstead Road, London NW1 7QY, UK; 2 Prescribing Observatory for Mental Health, Royal College of Psychiatrists Centre for Quality Improvement, 4th Floor, Standon House, 21 Mansell Street, London E1 8AA, UK; 3 Centre for Mental Health, Division of Experimental Medicine, Imperial College, Charing Cross Campus, St. Dunstan's Road, London W6 8RP, UK; 4 National Patient Safety Agency, 4-8 Maple Street, London WIT 5HD, UK

## Abstract

**Background:**

Lithium is a commonly prescribed drug with a narrow therapeutic index, and recognised adverse effects on the kidneys and thyroid. Clinical guidelines for the management of bipolar affective disorder published by The National Institute for Health and Clinical Excellence (NICE) recommend checks of renal and thyroid function before lithium is prescribed. They further recommend that all patients who are prescribed lithium should have their renal and thyroid function checked every six months, and their serum lithium checked every three months. Adherence to these recommendations has not been subject to national UK audit.

**Methods:**

The Prescribing Observatory for Mental Health (POMH-UK) invited all National Health Service Mental Health Trusts in the UK to participate in a benchmarking audit of lithium monitoring against recommended standards. Data were collected retrospectively from clinical records and submitted electronically.

**Results:**

436 clinical teams from 38 Trusts submitted data for 3,373 patients. In patients recently starting lithium, there was a documented baseline measure of renal or thyroid function in 84% and 82% respectively. For patients prescribed lithium for a year or more, the NICE standards for monitoring lithium serum levels, and renal and thyroid function were met in 30%, 55% and 50% of cases respectively.

**Conclusions:**

The quality of lithium monitoring in patients who are in contact with mental health services falls short of recognised standards and targets. Findings from this audit, along with reports of harm received by the National Patient Safety Agency, prompted a Patient Safety Alert mandating primary care, mental health and acute Trusts, and laboratory staff to work together to ensure systems are in place to support recommended lithium monitoring by December 2010.

## Background

Lithium is licensed for the acute treatment of mania, prophylaxis in bipolar disorder and to augment antidepressants in treatment-refractory recurrent depression. Its use for these indications is supported by contemporary UK treatment guidelines [1-3]. For most patients, treatment with lithium is long term [4].

Lithium is generally ineffective when the serum level is below 0.4 mmol/L, and very few patients will benefit from levels greater than 1.0 mmol/L [5]. Increasing levels above this upper threshold are associated with signs and symptoms of lithium toxicity such as confusion, seizures and renal damage. Treatment guidelines therefore recommend that the serum lithium level should be checked regularly throughout treatment to ensure that it remains within the therapeutic range. With regard to the frequency of monitoring, the NICE guideline for bipolar disorder [1] recommends that serum lithium is checked every 3 months while the British Association for Psychopharmacology guidelines for bipolar disorder [3] recommend every 3-6 months.

The side-effect profile of lithium is well established. As lithium is almost wholly excreted in the urine, any changes in renal function or fluid balance caused by intercurrent illness or drug treatment can potentially lead to lithium accumulation, which in turn can lead to renal damage and toxicity. Lithium treatment also increases the risk of clinical hypothyroidism up to 5-fold, through complex mechanisms that are unrelated to dose [5]. These potential problems necessitate pre-treatment checks of renal and thyroid function, followed by regular checks for the duration of lithium treatment for all patients. The NICE guideline for bipolar disorder [3] recommends that renal and thyroid function tests are conducted every 6 months while the BAP guideline for bipolar disorder [1] recommends that this biochemical monitoring is carried out every 12 months. In addition, lithium treatment is associated with weight gain [6] and NICE recommends that patients receiving lithium should have their body weight, BMI or waist circumference measured at least annually [3].

In the UK, the Quality and Outcomes Framework (QOF) also sets targets for the monitoring of patients receiving lithium in primary care [7]. QOF targets are less strict than those recommended by NICE. The data collected for each practice are the proportions of patients receiving lithium who have had their thyroid and renal function checked in the previous 15 months (mental health standard 4) and have had a serum lithium level within the therapeutic range documented in the previous 6 months (mental health standard 5). Despite the existence of explicit standards for monitoring patients who are prescribed lithium, a number of local audits conducted in different areas of the UK over the last 20 years have found this monitoring to be suboptimal [8-11]. There are no published audits that are UK-wide or that post-date the publication of the NICE guideline for the management of bipolar disorder.

In 2009, 38 mental health Trusts in the UK participated in a baseline audit of the quality of lithium monitoring as part of a quality improvement program (QIP) run by the Prescribing Observatory for Mental Health (POMH-UK). The audit standards were derived from the recommendations in the NICE guideline for the management of bipolar disorder [3], and were as follows:

1: The following tests/measures should be undertaken before initiating treatment with lithium: (a) renal function tests; urea and electrolytes (U&Es) including creatinine (or e-GFR or creatinine clearance); (b) thyroid function tests (TFTs), and; (c) body weight or BMI or waist circumference

2: The following tests/measures should be conducted during maintenance treatment with lithium: (a) serum lithium level every 3 months; (b) renal and thyroid function tests every 6 months, and; (c) weight or BMI or waist circumference during the last year

We report on systems for managing lithium treatment within these 38 mental health Trusts, and on how lithium monitoring compared with the standards set by NICE and the targets set by the QOF.

## Methods

The Prescribing Observatory for Mental Health (POMH-UK) conducts clinical audit-based QIPs that focus on prescribing practice in mental health. Each QIP starts with a baseline audit of practice against evidence-based clinical standards, and this is followed by the delivery of a benchmarked audit report, the provision of change interventions and finally a re-audit 12-18 months later. Further information about POMH can be found at www.rcpsych.ac.uk/pomh.

### The sample

POMH-UK invited all National Health Service (NHS) Trusts in the United Kingdom providing specialist mental health services to participate in a QIP focusing on the quality of monitoring of patients prescribed lithium. This was done in a number of ways which included; (1) e-mail communication with the POMH leads in eligible Trusts; (2) discussion with clinicians and clinical audit staff at POMH regional workshops, and: (3) letters of invitation to Trust chief executives, medical directors, chief pharmacists and clinical governance leads. Thirty-eight Trusts chose to participate. Very few UK services have a central register of patients prescribed lithium and Trusts used a variety of methods to identify their sample. These included a census of prescriptions, pharmacy records, pathology records and the caseloads of individual clinical teams. Services could enter data for as many patients as they wished.

### Data collection

For each patient the following data were collected: age, gender, ethnicity, and primary psychiatric diagnosis.

For the subsample of patients who had started lithium treatment in the past year, the following data were collected: the presence of documented pre-treatment measures of renal and thyroid function and body weight (or BMI or waist circumference), and documented evidence that the patient had been advised of the side effects of lithium, the risk factors for lithium toxicity and the signs and symptoms of toxicity.

For the remaining patients, who had been prescribed lithium for longer than year, the data collected included the number of occasions on which a serum lithium level, renal and thyroid function tests and a measure of body weight had been measured over the past 12 months. Multiple tests conducted within the same month were counted as a single test as these were likely to have been conducted for a purpose other than routine monitoring.

For each patient, all the data were collected from their clinical records, and submitted to POMH using a secure web-based system called SNAP. Data collection fields relevant to the audit standards were mandatory in that it was not possible to submit data for cases where the mandatory fields had not been completed. The identity of each Trust submitting data was known to POMH, but the identities of the individual clinical teams and patients were not. Only the national level data are reported here.

### Trust Questionnaire

Each Trust was sent a questionnaire relating to systems for managing patients who were prescribed lithium, both within the Trust and across the interface with primary care. With respect to systems within the Trust, the questionnaire covered whether: (1) there were locally adopted guidelines for managing patients prescribed lithium; (2) Trust clinicians had electronic access to pathology results; (3) care was wholly or partly delivered through lithium clinics and; (4) whether the Trust had access to an electronic database containing details of all patients prescribed lithium, and if so, whether this system generated automatic reminders that blood tests were due. Further questions were asked about systems for sharing care between the mental health Trust and primary care.

### Statistical analysis

Each of the four outcomes of interest (measures of serum lithium level, renal function, thyroid function and body weight) was treated as a binary measure; whether or not the standard had been met. Logistic regression analyses were conducted to explore the contribution of several possible explanatory variables (age, gender, ethnicity, psychiatric diagnosis, and care provider) to these binary outcomes. The separate effect of each predictor variable upon each outcome was tested in a series of univariable analyses. Subsequently, the joint effect of the variables upon each outcome was examined in multivariable analyses, using a backwards selection procedure to retain only the statistically significant variables. Data were analysed using SPSS, version 17.

## Results

### The sample

Four hundred and thirty six clinical teams from 38 mental health Trusts submitted data for 3,373 patients. 1,972 (59%) patients were female, 2,667 (79%) were white British, and the mean age of the sample was 55 years (sd 16, range 17-94 years). For 1,919 (57%) patients the primary clinical diagnosis was bipolar disorder, 857 (25%) unipolar depression, 370 (11%) a psychotic spectrum disorder (ICD10 F20-29), 161 (5%) another psychiatric diagnosis, and for 66 (2%) no psychiatric diagnosis was documented.

### Performance against the standards in the sub-sample of patients who had been prescribed lithium for less than 1 year

397 patients had been prescribed lithium for less than 1 year. Of these, 334 (84%) had a documented baseline test of renal function including creatinine; the respective figures for thyroid function and body weight were 325 (82%) and 145 (37%).

With respect to documentation regarding the provision of relevant information to patients, this was present for the side effects of lithium in 244 (62%) cases, the risk factors for toxicity in 166 (42%), and the signs and symptoms of toxicity in 178 (45%) of cases. These proportions did not differ for the sub-groups of patients who were either younger than 65 years or older than 65 years.

### Performance against the standards in the sub-sample of patients who had been prescribed lithium for more than a year

2,976 patients had been prescribed lithium for more than a year. With respect to lithium serum levels, 68% of cases had 2 or more documented tests in the previous year, thus meeting the QOF standard, while 30% had 4 or more tests in the last year, reaching the NICE standard. With respect to tests of renal function, which included creatinine, 81% of cases had one or more documented tests in the last year, thereby meeting the QOF standard, while 55% had two or more documented tests and therefore met the NICE standard. The respective figures for thyroid function were 82% and 50%. For 206 (7%) patients there was no documented evidence that any of the recommended monitoring tests/measures had been conducted in the previous year.

Further details of performance against the NICE and QOF standards are shown in Table 1. The summary results can be compared with those of previous published UK audits in Table 2. Table 3 provides further information on the demographic and clinical characteristics of the subsample of patients who been prescribed lithium for a year or more. It also indicates the relationship between each these variables and the extent to which the audit standards derived from the NICE guidance were being met.

**Table 1:** Lithium monitoring tests or measures conducted during maintenance treatment (n = 2,976)

Number of tests in last year	U&Es with creatinine	Thyroid function tests	Weight/BMI/ waist circumference	Serum lithium
**0**	**553 (19%)**	**524 (18%)**	**2155 (72%)**	**273 (9%)**

**1**	***795 (27%)***	***976 (33%)***	416 (14%)	**668 (22%)**

**2**	592 (20%)	693 (23%)	155 (5%)	***572 (19%)***

**3**	466 (16%)	453 (15%)	90 (3%)	***561 (19%)***

**4**	313 (11%)	208 (7%)	62 (2%)	503 (17%)

**5 or more**	257 (9%)	122 (4%)	98 (3%)	399 (13%)

Bold text Neither NICE standards nor QOF targets met

Bold and italics Meets QOF targets, but not NICE standards

Normal text Meets both QOF targets and NICE standards

**Table 2:** Results of prior, published UK audits of lithium monitoring

Study	Number of patient records audited	Mean age: years	% female	% with a diagnosis of bipolar disorder	% meeting standard relating to monitoring lithium level	% meeting standard relating to monitoring renal function	% meeting standard relating to monitoring thyroid function	Standards used in audit
**Current study**	**2,976**	**55**	**59**	**57**	**30** **68**	**55** **81**	**50** **82**	**NICE** **QOF**
Kehoe & Mander 1992 ^9 ^(Edinburgh)	458	56	68	56	< 81	-	-	BNF
*Eagles et al 2000 ^11^ (Aberdeen)	422 403	- 54	- 63	- -	54 54	71 78	44 55	BNF
Ryman 1997 ^34^ (Gateshead)	290	56	-	-	69	67	47	BNF
Fielding et al 1999 ^10^ (Southampton)	246	79	72	18	84	84	84	BNF
Head 1998) ^23^ (Cambridge)	148	65-87	76	56	36	74	80	BNF
+Farooqi et al 2002 ^13^ (Leicestershire)	92 122	- -	- -	- -	43 57	42 62	59 61	BNF
Glover & Lawley 2005 ^8 ^(Hull)	?50	-	-	-	52	66	64	BNF

*Comparison of monitoring practice before and after the distribution of monitoring guidelines

+Comparison of monitoring practice before and after the introduction of a local register

**Table 3:** Effect of patient characteristics on monitoring quality (NICE standards)

		n(%) of all patients:	n (%) of patients in each demographic or clinical group meeting NICE monitoring standards for:			
			Lithium levels	Renal function (Cr)	Thyroid function	Body weight
**Sex**	Male	1270 (42.7%)	363 (28.6%)	703 (55.4%)	600 (47.2)	371 (29.2%)
	Female	1706 (57.3%)	539 (31.6%)	925 (54.2%)	876 (51.3%)	450 (26.4%)
						
**Age**	< 65	2068 (69.5%)	587 (28.4%)	1060 (51.3%)	1000 (48.4%)	599 (29.0%)
	> 65	908 (30.5%)	315 (34.7%)	568 (62.6%)	476 (52.4%)	222 (24.4%)
						
**Ethnicity**	White British	2356 (79.2%)	709 (35.1%)	1264 (53.7%)	1162 (49.3%)	650 (27.6%)
	Black British	79 (2.7%)	18 (22.8%)	45 (57.0%)	42 (53.2%)	31 (39.2%)
	Asian	118 (4%)	32 (27.1%)	70 (59.3%)	58 (49.2%)	43 (36.4%)
	Other	53 (1.8%)	13 (24.5%)	30 (56.6%)	30 (56.6%)	22 (41.5%)
	Not stated	370 (12.4%)	130 (35.1%)	219 (59.2%)	184 (49.7%)	75 (20.3%)
						
**ICD code**	F20-29	326 (11%)	97 (29.8%)	182 (55.8%)	151(46.3%)	127 (39.0%)
	F30-39	2451 (82.4%)	753 (30.7%)	1349 (55.0%)	1245 (50.8%)	623 (25.4%)
	Other	137 (4.6%)	27 (19.7%)	59 (43.1%)	50 (36.5%)	62 (45.3%)
	Not known	62 (2.1%)	25 (40.3%)	38 (61.3%)	30 (48.4%)	9 (14.5%)
						
**Care provider**	General adult service	2155 (72.4%)	621 (28.8%)	1141 (52.9%)	1081 (50.2%)	549 (25.5%)
	Older peoples service	568 (19.1%)	220 (38.7)	374 (65.8%)	309 (54.4%)	142 (25.0%)
	Forensic service	76 (2.6%)	36 (47.4%)	60 (78.9%)	43 (56.6%)	56 (73.7%)
	Learning disabilities	136 (4.6%)	22 (16.2%)	38 (27.9%)	34 (25.0%)	60 (44.1%)
	Other service	41 (1.4%)	3 (7.7%)	14 (35.8%)	9 (23.1%)	12 (30.8%)

### Factors predicting monitoring performance

The univariable analyses examined the effect of potentially relevant clinical or demographic factors (age, gender, ethnicity, ICD-10 psychiatric diagnosis and type of clinical service providing care, e.g. general adult psychiatry, learning disability, forensic service, etc.) on whether the four outcomes were met. At a significance level of p ≤ 0.001, age (being over 65 years) and service type (elderly mental health services) were associated with monitoring of serum lithium level and renal function, service type (again essentially elderly mental health services) was associated with monitoring of thyroid function, while diagnosis (schizophrenia spectrum disorder) and service type (forensic and learning disability services) were associated with measurement of body weight.

The multivariable analyses addressed biochemical monitoring, and revealed that only service type (elderly mental health services) was associated with meeting the standards for monitoring serum lithium (OR 1.34; 95% CI 1.13-1.58) and renal function (OR 1.45; 1.12-1.87), both at a significance of p ≤ 0.001.

### Trust questionnaire

All 38 Trusts returned a completed questionnaire. Twenty-eight (74%) Trusts reported having fully adopted formal guidelines; most using the monitoring standards recommended in the NICE bipolar guidelines (n = 20) or British National Formulary (n = 11). Twenty-four (63%) Trusts reported having at least one lithium clinic, but only 8 (21%) had Trust-wide electronic access to results and 1 (3%) a local electronic database specifically for lithium that automatically produced prompts when tests were due. Fourteen (37%) Trusts had formally agreed, shared-care guidelines for patients managed concurrently with primary care, and 5 (13%) had electronic systems shared Trust-wide between primary and secondary care.

## Discussion

The main findings were that documented evidence that baseline tests of renal and thyroid function had been conducted was found for just over four-fifths of patients recently commenced on lithium therapy, and for those patients receiving lithium treatment for a year or more, the frequency of monitoring of serum lithium and renal and thyroid function met the standards set by NICE in less than a third to just over a half of patients, depending on the measure.

Previous published audits of the quality of lithium monitoring have tended to be relatively small and locality specific. They also pre-date the NICE bipolar guideline, and used older audit standards from the British National Formulary (see table 2). These factors render it difficult to directly compare our findings with those of the audits conducted earlier in this area, but there is little to suggest a trend for improvement over time.

### Why is recommended monitoring not carried out?

Possible explanations for suboptimal monitoring may implicate procedural, patient and/or practitioner variables.

#### Procedural factors

With respect to procedural factors, previous audits have reported incomplete local implementation of monitoring guidelines [11], poor communication of test results to clinical teams, lack of communication between primary and secondary care [12] and a lack of dedicated monitoring services and central registers that generate reminders that tests are due [10,13]. Our study corroborates these findings by revealing variable adoption of monitoring guidelines and use of shared care protocols by mental health Trusts, with few clinicians having electronic access to test results. In addition, few Trusts operate designated lithium clinics and only one reported having a local database specifically for lithium that produced automatic prompts when biochemical tests were due.

Communication could be improved through the development of local registers of lithium-treated patients (with systems for review and recall), and local needs assessment (complemented by audit, training and the use of appropriate guidelines) [12-14]. Bringing primary and secondary care teams together to agree on a model of shared care suited to local needs may also be important [14].

#### Patient factors

Previous studies have identified a number of patient-related factors that may influence monitoring rates. These include variation in the willingness of patients to have blood tests [15,16], and either receiving inadequate information about lithium treatment and the need for regular blood tests or not assimilating the information given [8,17,18]. Our findings provide support for the view that many patients are not provided with basic information about their lithium treatment.

Patient demographics may plausibly influence the quality of monitoring of psychotropic medication [19], but to what extent this would be driven by variable engagement with healthcare by patients and the behaviour of clinicians is uncertain. Our study did not identify any contribution from gender or ethnicity, but found that monitoring practice for patients cared for by older peoples services was generally better than that provided by general adult services. This may reflect that clinicians in elderly services have an increased awareness of lithium monitoring requirements for their patients, who are particularly vulnerable to renal side-effects, and in whom the background prevalence of thyroid problems is higher than in younger adults. Our audit also revealed slightly superior monitoring of body weight for patients with a diagnosis of a schizophrenia spectrum disorder, which may indicate increased clinician awareness of risk factors for weight gain in such patients [20,21].

#### Practitioner factors

With respect to practitioner-related factors, several studies report superior standards of lithium monitoring for patients under the care of a psychiatrist [9,11,22] while others report no difference from the quality of monitoring undertaken by general practitioners [23,24]. Some audits also report superior monitoring for those patients in nurse-led, designated lithium clinics [10] or under pharmacist supervision [25]. It has been suggested that the large variation in the degree of knowledge about lithium and its monitoring requirements amongst individual professionals may account for these inconsistent findings [26]. There may also be variation between clinicians in the acceptance of the need for monitoring at the frequency recommended by NICE.

### The use of incentivised care in improving monitoring practice

Mental health Trusts within the UK are required to implement NICE guidelines and progress with this is monitored by the Care Quality Commission (CQC). In contrast, there are no sanctions for General Practitioners who fail to meet QOF targets, rather a positive benefit in the form of payment when these targets are met.

In our sample, the primary care QOF targets with respect to monitoring of serum lithium was met in over two-thirds of cases, and the target with respect to renal and thyroid function in just over four fifths. The NHS Information Centre (QOF statistics for England, 2008/9) lists these targets as having been met for 91% and 97.4% of patients respectively within primary care in 2008/9 [27]. As it is likely that the care of the majority of patients who are prescribed lithium is shared between primary and secondary care, less apparent monitoring in our secondary care sample may partly reflect communication issues between these sectors.

Proponents of a system like QOF argue that it can improve the implementation of evidence-based interventions [28] in primary care and constitutes an important quality improvement tool. However, critics have expressed concerns that QOF targets are too low with poor discriminatory value [29], and that incentivised care will never be an adequate substitute for professional judgment [30]. Our finding that the proportion of patients monitored in line with QOF targets was higher in primary than secondary care supports the view that the QOF system is a viable quality improvement tool. There is however, a need for more objective and transparent setting of QOF targets and increasing convergence between these and NICE standards.

### Study strengths and limitations

A possible limitation of our study is a bias in the selection of patient samples for audit by each participating Trust. Such bias is unlikely to be unidirectional in that clinical teams that consider they are performing well in relation to meeting the relevant practice standards may choose to participate, whereas Trusts may choose to submit data for teams that they suspect are performing less well. The net result of competing sources of bias is unknown. Poor documentation standards or quality of case note review in this audit could also account for observed failures in monitoring practice.

A strength of the work is that our audit sample is larger than those of all previously published studies combined and is drawn from across the UK. Trusts that participated in the audit are representative of all NHS mental health Trusts [31] and so it is likely that our findings are generalisable to practice in other Trusts and representative of current clinical practice in the UK.

## Conclusions

This is the first, published, national-level audit of lithium prescribing and monitoring practice in the UK. Our findings suggest that contemporary lithium monitoring falls short of the standards recommended by NICE. Failure to provide adequate information to ensure the safe use of lithium and/or to ensure adequate monitoring of established treatment, may place patients at risk of avoidable drug related morbidity.

The National Patient Safety Agency (NPSA) is a special health authority that was established in 2001. Its role is to co-ordinate information about harm caused in health care settings, and to work with partner organisations to reduce such harm. Partly in response to the findings from this audit and partly in response to reported patient safety incidents related to lithium, the NPSA issued a Patient Safety Alert with actions requiring that primary care, mental health and acute Trusts, along with hospital pathology services ensure systems are put in place to support the monitoring associated with lithium treatment that is recommended by NICE [32]. The NPSA has also endorsed a patient-held pack which contains information about treatment including how to avoid toxicity, and a biochemical monitoring record [33,34]. The deadline for getting information to patients and having these monitoring systems in place is December 2010.

## Competing interests

C.P and T.B. have acted as consultants to pharmaceutical companies marketing antipsychotic medication; NC, AS-S and DH have nothing to declare.

## Authors' contributions

NC: conducted the literature search, contributed to the design of the study, reviewed the data and contributed to drafting the paper. TREB: contributed to the literature search, the design of the study, analysis and interpretation of the data, and writing the paper. ASS: contributed to the design of the study, co-ordinated data collection and analysis, and contributed to drafting the paper. DG: contributed to the methodology of the study, interpretation of the data and drafting the paper. CP: contributed to the literature search, the design of the study, analysis and interpretation of the data, and writing the paper, and is the guarantor for this paper. All authors read and approved the final manuscript.

## Pre-publication history

The pre-publication history for this paper can be accessed here:

http://www.biomedcentral.com/1471-244X/10/80/prepub
